# Green tea consumption rapidly enhances cognitive performance and flow state during mental tasks in healthy young adults

**DOI:** 10.1371/journal.pone.0328394

**Published:** 2025-07-10

**Authors:** Chie Kurosaka, Shinji Miyake, Makoto Kobayashi, Chika Tagata, Yuka Tatsumi

**Affiliations:** 1 Department of Human, Information and Life Sciences, School of Health Sciences, University of Occupational and Environmental Health, Kitakyushu, Fukuoka, Japan; 2 Graduate School of Science and Technology, Chitose Institute of Science and Technology, Chitose, Hokkaido, Japan; 3 Central Research Institute, ITOEN, Ltd., Makinohara, Shizuoka, Japan; UniEVANGELICA University Centre of Anapolis: UniEVANGELICA Centro Universitario de Anapolis, BRAZIL

## Abstract

This study examined green tea’s effects on task performance, mental fatigue, workload, and flow experience. Twenty-two healthy young male participants (mean ± SD age = 21.86 ± 1.96 years, range = 18–26 years) completed two 5-min mental tasks (arithmetic and sequential digit search) under three beverage consumption conditions: no beverage (NONE), WATER, and TEA (green tea). In the WATER and TEA conditions, participants consumed 70 mL of beverage three times during the session (total: 210 mL). Subjective Fatigue Feelings was assessed before and after each condition, and subjective evaluations including NASA Task Load Index, Flow Experience Checklist, and Duration Judgment Ratio, were conducted after each task. Results showed no significant differences in task performance across conditions (p > 0.05, η² = 0.11 and η² = 0.02 for MATH and SDS tasks, respectively), likely due to ceiling effects (accuracy > 95%). In the TEA condition, arousal levels were maintained and no increase in mental fatigue was observed, unlike the other conditions. The flow state score in the TEA condition was significantly higher than that in other conditions (NONE vs TEA: p = 0.0009), and participants perceived shorter task duration (NONE vs TEA: p = 0.0016, WATER vs TEA: p = 0.0061). These effects were observed even with small amounts of beverage and short consumption periods, suggesting that habitual intake of low-dose green tea may enhance task engagement and flow experience in everyday tasks.

## Introduction

Green tea is made from the leaves of *Camellia sinensis* and is among the most widely consumed beverages worldwide. Green tea contains catechins, caffeine, theanine, and vitamins, which have many health benefits. The consumption of green tea has also been suggested to reduce the risk of developing diabetes [1] and may lower the risk of colorectal and gastric cancers [2–4], making it a promising contributor to the maintenance of health. These findings contribute to the increasing global popularity of Japanese green tea as a preferred beverage choice. L-theanine, an amino acid abundant in green tea, has gained attention owing to its potential to counter caffeine-induced excitation and promote relaxation [5]. The oral ingestion of L-theanine by female university students leads to the production of alpha waves in the occipital and parietal regions of the brain, suggesting a potential relaxation effect [6]. Additionally, a study investigating the effects of L-theanine consumption at realistic dietary levels indicated that the increase in alpha wave activity was consistent across sexes, reinforcing its role in promoting relaxation and mental clarity [7]. Kimura et al. also reported that L-theanine reduced both psychological and physiological stress responses in healthy participants during a mental arithmetic task [8]. L-theanine combined with caffeine enhances performance in high-load attention-switching tasks; this combination contributes to self-reported alertness and reduces self-reported tiredness [9], consistent with previous findings [10,11]. Beyond the effects of these components, the distinctive aroma of green tea induces positive effects, such as anti-stress, relaxed feelings, improved task performance, and positive responses in electroencephalogram activity and salivary chromogranin A (CgA) levels [12,13].

We have previously reported the effects of Japanese tea consumption on mental task performance. The results suggested that hojicha (roasted green tea) intake contributes to improved performance, reduced time pressure, and stable performance during a mental arithmetic task [14]. We also reported that the nosetip tissue blood flow measured by laser Doppler blood flowmeter and cortical oxygen consumption measured by near-infrared spectroscopy were lower in the tea condition than in the hot water condition [15]. These findings suggest that tea consumption may have beneficial effects not only on task performance and physiological responses but also on psychological states during task engagement. In this context, we are particularly interested in flow experience during office work, as it is considered a key factor in maintaining a sustained cognitive performance.

In relation to office work, “Good Health and Well-Being” stands out as a key Sustainable Development Goal (SDG), reflecting a worldwide effort to safeguard employee health and enhance work-life balance. Workers’ positive psychological condition is referred to as work engagement [16], which is defined as a positive, fulfilling, and work-related state of mind characterized by vigor, dedication, and absorption [17]. Absorption is characterized by being fully concentrated and deeply engrossed in one’s work, whereby time passes quickly and one has difficulty detaching from work. The state of complete absorption in this study was close to the flow state. Flow is more likely to occur when both an individual’s skill and challenge levels are high, leading to enhanced productivity and satisfaction [18]. In sports, this is often referred to as being “in the zone,” and it is believed that experiencing this state maximizes cognitive abilities and enhances skills [19].

Csikszentmihalyi [20] proposed the concept of flow, which describes a state of deep absorption in which individuals lose track of time. Subsequent research categorized mental states into eight distinct types based on the balance between perceived challenges and skill levels. Although some studies on flow have been conducted in sports [21,22], data on other activities are limited. However, flow can be experienced in various contexts, including enjoyable activities such as playing the piano, gaming, reading, working, and academic learning [18,23]. Therefore, even during routine office work, when individuals are engaged in their tasks and experience heightened productivity, they can be considered to be in a flow state. This is supported by previous findings indicating that a flow state was induced using a mental task modeled on office work based on a field survey of desk workers [24]. Reich et al. reviewed whether caffeine can act as a chemical facilitator of flow states and concluded that caffeine may enhance flow under cognitively demanding conditions [25]. Despite this, few studies have examined the occurrence of flow during routine cognitive tasks such as office work, and few studies have evaluated the potential of functional beverages, such as green tea, in promoting this optimal mental state. In light of these considerations, we hypothesized that acute green tea consumption would reduce the perception of mental fatigue and enhance subjective flow experience.

In this study, we investigated the relationship between green tea consumption, task performance, and flow state during mental tasks. This study is one of the first to investigate the acute impact of consuming a small dose of green tea on inducing flow state and maintaining vigilance during short-term mental tasks in a controlled environment using combined subjective and objective measures.

## Materials and methods

### Participants

Participants with no history of gastritis or stomach pain after consuming green tea and who were not prone to diarrhea or other symptoms after drinking water were recruited. Although physiological measurements were also conducted in this study, their analysis is beyond the scope of this paper and will be reported in a separate study. Accordingly, participants were excluded from the experiment if they (1) had circulatory problems, such as arrhythmia, or were taking medication, (2) smoked, or (3) had dry skin, such as atopic dermatitis. The menstrual cycle has been shown to affect task performance, with a decline observed premenstrually [26,27]. Emma et al. [28] conducted a meta-analysis of 241 million observations from 3.3 million women across 109 countries. Their study demonstrated that, compared to daily, weekly, and seasonal cycles, the menstrual cycle exerts the greatest influence on most measured dimensions of mood, behavior, and vital signs. The choice of a sample composed exclusively of young men aimed to control physiological variables such as hormonal fluctuations related to the menstrual cycle, which can affect the state of wakefulness, perception of mental fatigue, and physiological measurements. The recruitment period for this study was from December 1, 2022, to March 15, 2023. A total of 22 healthy males (Age: mean ± SD = 21.86 ± 1.96 years, range = 18–26, Height: mean ± SD = 173.3 ± 5.39 cm, range = 165–186, Weight: mean ± SD = 66.7 ± 8.28 kg, range = 50–80) provided written informed consent after receiving a written description of the study and voluntarily participated in the study. The participants were instructed to abstain from caffeine intake until the start of the experiment and to ensure adequate sleep the night before. This study was approved by the Ethics Committee of the University of Occupational and Environmental Health, Japan (Approval no. R4-055, November 11, 2022) and was performed in accordance with the relevant guidelines and regulations.

## Study design

There are two approaches to evaluating the effects of green tea: direct and indirect evaluations. The direct evaluation examines the physiological responses induced by tea consumption, such as increased parasympathetic nervous activity, and subjective reactions, such as enhanced feelings of relaxation. However, physiological responses caused by such subtle factors as green tea intake are often weak and difficult to detect. Therefore, an experimental approach was employed that involved the deliberate induction of psychological stress, and the effect of tea consumption was assessed by comparing the differences in responses between stress conditions. This approach is referred to as an indirect evaluation. In this method, task performance on cognitive stressors can also be assessed, allowing for the examination of the effects on cognitive function enhancement. This study used an indirect evaluation approach to address the research objectives.

### Procedure

**Fig 1:**
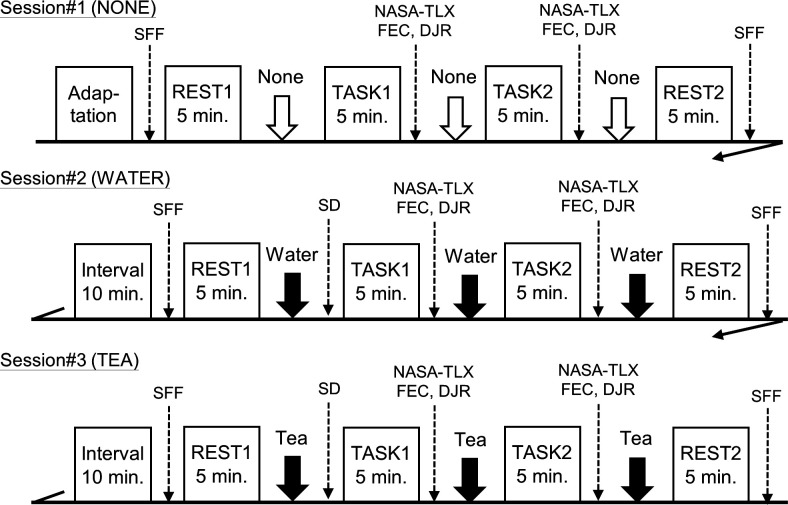
Experimental procedure. *Notes:* SD: Evaluation of drink by semantic differential method, SFF: subjective fatigue feelings (“Jikaku-sho shirbe”), NASA-TLX: NASA task load index, FEC: Flow checklist, DJR: Duration judgment ratio.

Participants practiced the mental task after checking the experimental procedure and instructions for the mental task. The experimental procedure is illustrated in Fig 1. They stayed calm for 10 minutes to adapt to the experimental situation. After adaptation, the Subjective Fatigue Feelings (SFF) questionnaire was administered. Subsequently, the participants remained calm for 5 min, consumed water or green tea, and evaluated their taste and aroma using the semantic differential (SD) method. No beverage was consumed under control conditions (first session: NONE). They then performed two mental tasks, a mental arithmetic task and a sequential digit search, for 5 min, followed by another 5 min resting period. After each task, participants assessed subjective evaluations using the NASA Task Load Index (NASA-TLX), Flow Experience Checklist (FEC), and Duration Judgment Ratios (DJR) and consumed a beverage again. This procedure was repeated three times successively with 10 min intervals under two different beverages and a control condition. In the second session, the participants consumed water (WATER); in the third session, they consumed green tea (TEA). All three sessions (NONE, WATER, and TEA) were conducted on the same day following the same experimental procedure. In the WATER and TEA conditions, 70 mL of the beverage was served in paper cups, and the participants consumed it three times, totaling 210 mL for each beverage condition. After completing all sessions, the participants reported their tasks (likes or dislikes), strengths or weaknesses of the tasks, and their usual beverage consumption habits. Counterbalancing the order of conditions is a standard method to avoid learning effects. However, in this study, owing to the residual components of tea, it was not possible to counterbalance the order of the three beverage conditions, and all participants underwent the sessions in the fixed order of NONE, WATER, and TEA conditions. Throughout the experiment, each participant was alone in the soundproof measurement room and performed the tasks according to the experimenter’s instructions, who was monitored from the next room. Subjective evaluations and mental tasks were displayed on a 27-inch computer monitor placed in front of the participants, and every operation was performed using only a mouse. All experiments were conducted in the Psychophysiology and Ergonomics Research Laboratory at the University of Occupational and Environmental Health, Japan.

### How drinks were provided

The beverages used were water and green tea, and were divided into four lidded paper cups (70 mL each); one cup was used to measure the temperature of the beverage. The temperature of the beverages was almost stable throughout the session and staying at 25 ± 1 °C, while the room temperature ranged from 23 to 27 °C. All cups were placed in front of the participants, and they consumed the beverages within 45 seconds while watching an analog timer displayed on a PC screen. Given that this study focused on the effects of the daily consumption of commercially available green tea, the doses of components such as catechins and L-theanine were lower than those in previous studies [7,29,30]. The results of the beverage component analysis conducted by the Japan Food Research Laboratories and Showa Denko Materials Techno-Service Corporation are shown in S1 Table. The aroma components, such as 2-Ethyl-3, 5-dimethylpyrazine, tetramethylpyrazine, and 2,3-diethyl-5-methylpyrazine, were analyzed by dynamic headspace-gas chromatography (DHS-GC) -/MS using a Tenax TA trapping system (GERSTEL GmbH & Co., KG, Germany). A GERSTEL MPS autosampler (GERSTEL GmbH & Co., KG), an Agilent 7890 B GC system (Agilent Technologies, Santa Clara, CA, USA), and an Agilent 5977A mass spectrometric degradation system (Agilent) were used. The analytical column was DB-WAX (60 m × 0.25 mm i.d.; d_f_ 0.25 μm; Agilent Technologies).

### Mental task

#### Mental arithmetic task (MATH).

A mental arithmetic task based on the MATH algorithm proposed by Turner et al. [31] was used. In this task, participants are presented with an arithmetic equation involving either addition or subtraction, followed by a numerical answer, and are required to judge whether the answer is correct or incorrect. The arithmetic equation is displayed on a computer screen for 2 s, followed by the word “EQUALS” for 1.5 s before the numerical answer is presented. The participants are instructed to respond by left-clicking if the answer was correct and right-clicking if the answer was incorrect. Regardless of accuracy, the next equation is presented immediately after their responses. The difficulty level of the arithmetic problems is categorized into five levels. The details of each difficulty level are listed in Table 1. Notably, the arithmetic problems used in this study differed from those originally proposed by Turner et al. [31]. In contrast to their machine-paced format, the present task was conducted in a self-paced manner. Additionally, all trials began at Level 3, and the next question level increased if the prior answer was correct and decreased otherwise. However, the level did not change if the participant made an error at Level 1 or answered correctly at Level 5. Each equation included a carry-over or a borrowing step. Participants were not informed about the progression of the difficulty level but received feedback on their response accuracy through a color-coded lamp on the screen (green for correct responses and red for incorrect responses).

**Table 1:** Mental arithmetic task level.

Level	Formula	Example
1 – easy	2-digit + 1-digit	34 + 7
2	2-digit - 1-digit	78 − 9
3	2-digit + or – 2-digit	65 + or – 28
4	3-digit + 2-digit	472 + 35
5 – difficult	3-digit - 2-digit	597 − 83

#### Sequential Digit Search Task (SDS).

This task originates from the Advanced Tail Making Task (ATMT), a computer software program developed as an improvement of the Trail Making Test (TMT), which is used to evaluate brain age and fatigue [32,33]. The SDS task is adapted from the ATMT to promote a flow experience by adding a 30-s progress bar and a hit indicator. The screen displays 30 randomly arranged three-digit numbers with one target three-digit number shown in the upper-right corner. Participants are required to search for the target number among 30 numbers and click on it. If they click an incorrect number, a red circle appears in the upper-left corner as feedback; if they select the correct number, the indicator turns green, and the next trial begins.

In this study, the initial target number was set to 523, and with each new trial, the target number increased sequentially, while the entire numerical arrangement was shuffled. To keep the task difficulty consistently high enough, the initial number was fixed at 523, and the system was configured so that the number did not transition to four-digit figures within the 5-min task duration. Additionally, the progress bar at the bottom of the screen gradually decreased over 30 s. Regardless of the participants’ responses, the numerical arrangement was reshuffled every 30 s. The task was self-paced; however, the participants were instructed to respond as quickly and accurately as possible.

#### Subjective Fatigue Feelings (SFF).

The subjective experience of fatigue was assessed using the SFF [34], a questionnaire developed by the Research Group of Industrial Fatigue under the Japan Society for Occupational Health. Designed to evaluate the degree of work-related fatigue, this instrument is regarded as a reliable tool for capturing workers’ subjective perceptions of fatigue. Participants rated 25 items [35], as listed in Table 2, on a five-point rating scale (1 = disagree completely, 2 = agree scarcely, 3 = agree slightly, 4 = agree considerably, and 5 = agree strongly). The scale comprises five factors: feeling of drowsiness, feeling of instability, feeling of uneasiness, feeling of local pain or dullness, and feeling of eyestrain, each comprising five items. The total SFF score was calculated as the sum of five scores, with a maximum possible score of 25.

**Table 2:** Factors of subjective fatigue feelings.

Factor	Fatigue feelings
**(I) Drowsiness**	I feel giving a yawn, I feel drowsy, I feel lack of a desire to do something, I feel tired in the whole body, I feel a desire to lie down
**(II) Instability**	I feel nervous, I feel restless, I feel anxiety, I feel depressed, I feel difficulty in thinking
**(III) Uneasiness**	I feel heavy in the head, I feel ill, I feel headache, I feel the brain hot or muddled, I feel dizziness
**(IV) Local pain or Dullness**	I feel stiff in the neck and shoulders, I feel a pain in the hands or fingers, I feel dullness in the arms, I feel a lower back pain, I feel tired in the legs
**(V) Eyestrains**	I feel dry eyes, I feel a pain in the eyes, I feel eyes blurred, I feel eyestrain, I feel eyes blinking

#### NASA Task Load Index (NASA-TLX).

The NASA Task Load Index (NASA-TLX) [36], a subjective measure designed to evaluate perceived workload, comprises six subscales: mental demand (MD), physical demand (PD), temporal demand (TD), own performance (OP), effort (EF), and frustration (FR). Furthermore, one item, overall workload (OA), was added. All participants were required to answer each subscale using a Visual Analog Scale (VAS), ranging from 0 to 100, to indicate low or high levels (poor or good for OP). The means of the six subscales (Raw TLX: RTLX), except for OA, and the Adaptive Weighted Workload (AWWL) were obtained. AWWL calculates weighted average values without pairwise comparisons and indicates a high correlation with the original weighted workload (WWL) score [37].

#### Flow Experience Checklist (FEC).

The flow state was assessed using the Flow Experience Checklist (FEC), a self-report measure that evaluates key dimensions of flow, including full concentration, altered perception of time, and deep immersion in the task. Given the six repeated measurements conducted in this study, a shortened 10-item version of the FEC, developed by Ishimura [38] based on the original 36-item Flow State Scale (FSS) [39], was employed to reduce participant burden and ensure data reliability. For the 10 items listed in Table 3, participants declared their state during the task using a 7-point scale (“Strongly agree = 7,” “Agree = 6,” “Slightly agree = 5,” “Neither agree or disagree = 4,” “Slightly disagree = 3,” “Disagree = 2,” “Strongly disagree = 1”). Ten items are grouped into three factors: “confidence in abilities (Confidence),” “strength of experience (Experience),” and “challenge toward the goal (Challenge)” and the average score for these three factors and the average of all 10 items (FLOW) were calculated.

**Table 3:** Items of flow experience checklist.

No	Item	Factor
**1**	I felt just the right amount of challenge.	Challenge
**2**	I was confident in managing matters	Confidence
**3**	I knew what I had to do each step of the way	Challenge
**4**	My thoughts/activities ran fluidly and smoothly.	Confidence
**5**	I had no difficulty concentrating.	Experience
**6**	The right thoughts/movements occurred of their own.	Confidence
**7**	I was completely lost in thought.	Experience
**8**	I felt that I had everything under control.	Confidence
**9**	I did not notice time passing.	Experience
**10**	I really enjoyed the experience.	Experience

#### Duration Judgment Ratio (DJR).

All participants were asked to rate their perception of the mental task duration compared with the actual time of 5 min [40]. Evaluation values were collected using the VAS method, with 0–100 points as integers.

### Analysis method

MATH performance was evaluated using the following indices: Correct Rate [100 × number of correct answers/total number of problems (%)], Number of Responses (total responses within the task duration of 5 minutes), Mean Reaction Time (msec) (calculated as 300,000/ Number of Responses), and Mean Level.

SDS performance was assessed using the Number of Total Clicks, Number of Correct Clicks, Correct Rate, Reaction Time (msec) (calculated as 300,000/ Number of Total Clicks), and Correct Reaction Time (msec) (calculated as 300,000/ Number of Correct Clicks).

For all performance and subjective evaluation indices, a paired *t*-test was repeated for multiple comparisons, and the significance level was adjusted using the Holm method. The mean total SFF score and the five factors were calculated for each beverage condition, and the scores at the beginning and end of each session were compared (paired *t*-test). The significance level was set at p < 0.05, p < 0.10 was considered to be marginally significant, and the statistical power (1-β) was calculated (G*Power 3.1.9.2). All data are expressed as mean ± standard error (SE), unless otherwise specified.

One participant was excluded because of unreliable responses across the six assessments. Specifically, this participant gave a rating of 100 (maximum score) on one occasion and 0 (minimum score) on the remaining five. Although these scores were not identified as an outlier by the Smirnov-Grubbs test [41], an MD score of 0 was considered unrealistic for tasks requiring mental and intellectual effort. Furthermore, inconsistencies similar to those found in the MD scores were also observed in other scores for this participant, leading to exclusion from the MATH and SDS tasks. In the SDS, one participant, excluded from the NASA-TLX analysis, rated all FEC items in the WATER condition as 1 (“not applicable at all”) and was determined to be an outlier using the same test (p = 0.0024); thus, was excluded. No other missing data were found.

All authors meet the authorship criteria as defined by the International Committee of Medical Journal Editors (ICMJE) and have approved the final version of this manuscript. Furthermore, to ensure scientific independence, the authors affiliated with the sponsoring company (ITOEN, Ltd.) were not involved in any part of the statistical analysis or data interpretation.

## Results

### Task performance

**Fig 2:**
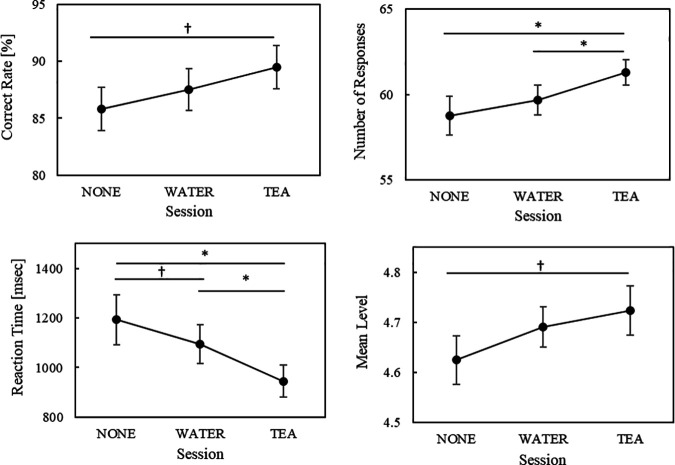
Task performance of MATH. *Notes:* Bars indicate the standard errors of the mean. † p < 0.1, * p < 0.05; MATH: mental arithmetic task.

**Fig 3:**
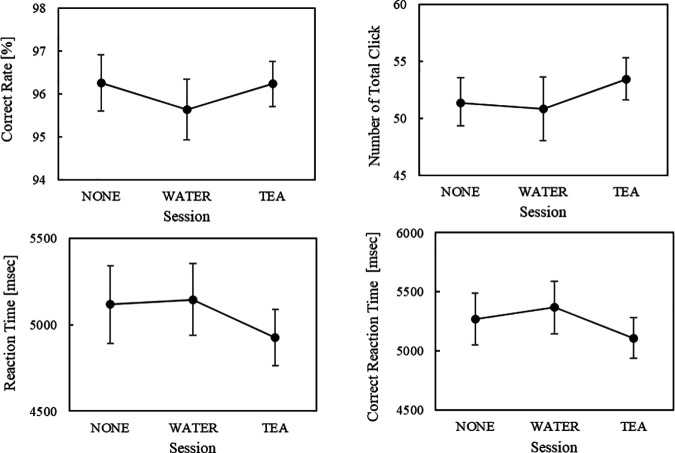
Task performances of SDS. *Notes:* Bars indicate standard errors of the mean; SDS: sequence digit search task.

The mean ± SE of the arithmetic task performance in the MATH for each condition is shown in Fig 2. The correct response rate was higher in the TEA condition than in the NONE condition (p = 0.099). The number of responses was significantly higher in the TEA condition than in the NONE (p = 0.0182, 1-β = 0.83) and WATER conditions (p = 0.0273, 1-β = 0.73). The reaction time was significantly shorter in the TEA than in the NONE condition (p = 0.0109, 1-β = 0.88) and significantly shorter in the TEA than in the WATER condition (p = 0.0378, 1-β = 0.68). Additionally, the reaction time in the WATER condition was shorter than that in the NONE condition (p = 0.0640), and the mean levels were higher in the TEA than in the NONE conditions (p = 0.0987). The mean ± SE values in the SDS for each calculated result are shown in Fig 3. No significant differences were observed between the sessions in any task performance (Fig 3). The descriptive statistics, including the mean, SD, n, and range for each task, are summarized in S2 and S3 Tables.

### Subjective assessment

#### SFF.

**Fig 4:**
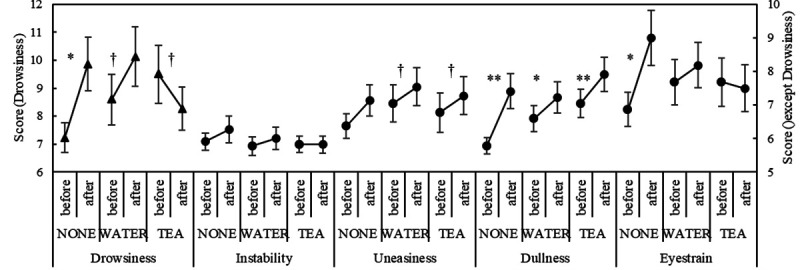
Results of the SFF score. *Notes:* † p < 0.1, * p < 0.05, ** p < 0.01; SFF: subjective fatigue feelings.

The SFF scores are shown in Fig 4. Drowsiness scores significantly increased after the task compared to before in the NONE (p = 0.002, 1-β = 1) and WATER conditions (p = 0.037, 1-β = 0.944). In contrast, in the TEA condition, no significant increase was observed; rather, the scores showed a slight decrease (p = 0.0693). There was no significant difference in uneasiness scores before and after the task in the WATER (p = 0.0856) and TEA (p = 0.0919) conditions, although a minimal numerical increase was noted. Dullness scores significantly increased after the task in all sessions compared to before (NONE: p = 0.005, 1-β = 0.93; WATER: p = 0.0157, 1-β = 0.71; TEA: p = 0.006, 1-β = 0.86). Eyestrain scores significantly increased after the task in the NONE condition (p = 0.0171, 1-β = 0.84). The summary of the SFF scores (mean, SD, n, and range) for each condition is shown in S4 Table.

#### NASA-TLX.

**Fig 5:**
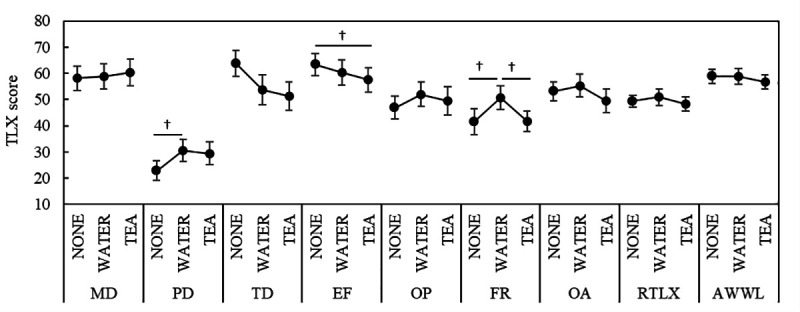
NASA-TLX scores in MATH. *Notes:* † p < 0.1; NASA-TLX: NASA task load index; MD: mental demand; PD: physical demand; TD: temporal demand; OP: own performance; EF: effort; FR: frustration; OA: overall workload; RTLX: mean of subscales; AWWL: adaptive weighted workload; MATH: mental arithmetic task.

**Fig 6:**
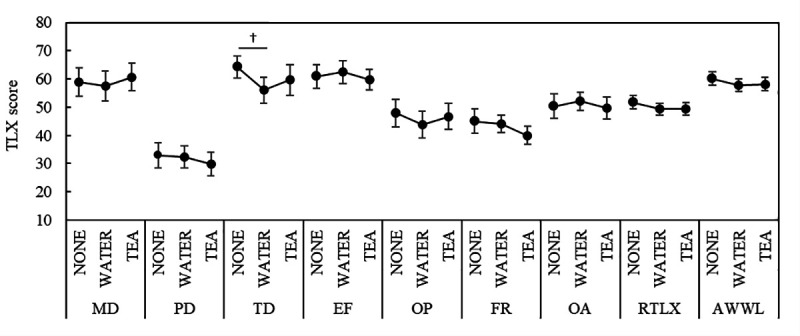
NASA-TLX scores in SDS. *Notes:* † p < 0.1; NASA-TLX: NASA task load index; MD: mental demand; PD: physical demand; TD: temporal demand; OP: own performance; EF: effort; FR: frustration; OA: overall workload; RTLX: mean of subscales; AWWL: adaptive weighted workload; SDS: sequence digit search task.

The results are shown in Fig 5 using MATH. Although not statistically significant, the PD score was higher in the WATER than in the NONE condition (p = 0.0606); the EF score was lower in the TEA than in the NONE condition (p = 0.0898); the FR score was higher in the WATER than in the NONE condition (p = 0.0926) and lower in the TEA than in the WATER condition (p = 0.0733). In the SDS task, the TD score was marginally significantly lower in the WATER than in the NONE condition (p = 0.0537); no other scores showed significant differences (Fig 6). The descriptive statistics for the NASA-TLX scores across tasks are summarized in S5 Table.

#### FEC and DJR.

**Fig 7:**
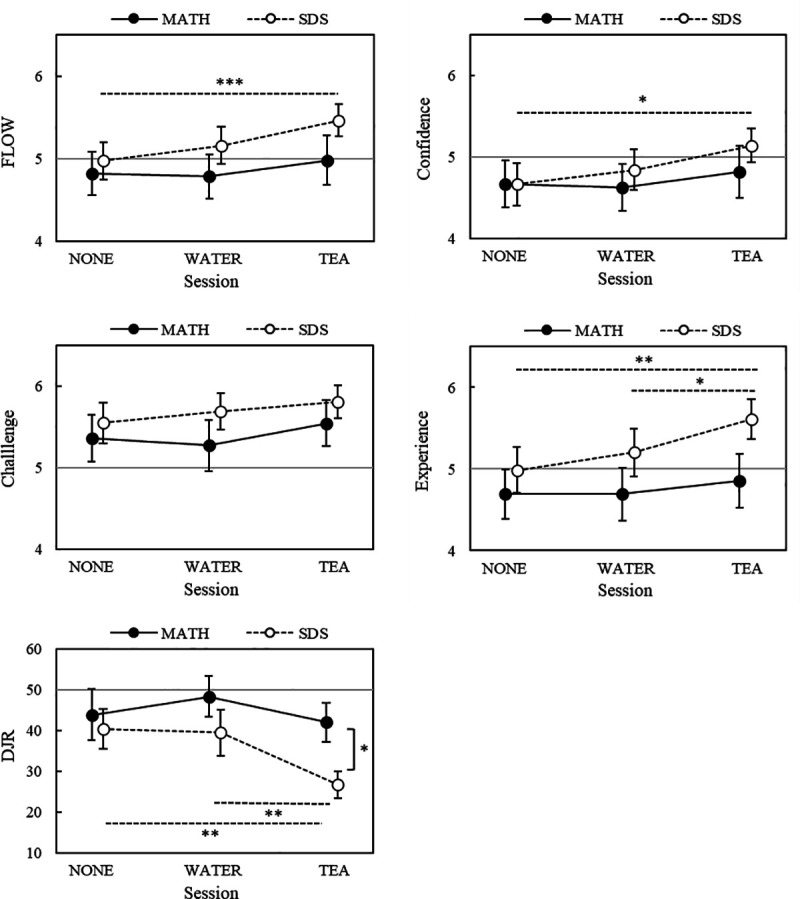
Comparison of FEC and DJR scores between the sessions and tasks. *Notes:* * p < 0.05,** p < 0.01, *** p < 0.001; FEC: flow experience checklist; DJR: duration judgement ratio; MATH: mental arithmetic task; SDS: sequence digit search task.

A comparison of the FEC scores and DJR for the two tasks is shown in Fig 7. In the MATH, no significant differences were observed between the sessions for any of the scales. As a result, the flow score and confidence score were significantly higher in the TEA than in the NONE condition (p = 0.0009, 1-β = 0.99; p = 0.0269, 1-β = 0.77). Additionally, the experience score in the TEA condition was significantly higher than that in the NONE (p = 0.0014, 1-β = 0.98) and WATER conditions (p = 0.0195, 1-β = 0.76). The perceived task duration during the SDS task in the TEA condition was significantly shorter than that in the NONE (p = 0.0016, 1-β = 0.97) and WATER conditions (p = 0.0061, 1-β = 0.89).

SDS had higher FLOW scale scores and lower DJR values than MATH. Paired *t*-tests with Holm’s correction were conducted for indicators with large differences between tasks in the TEA condition. The results showed that DJR (n = 21, p = 0.0014) was significantly lower for the SDS than for the MATH. S6 Table presents the descriptive statistics of the FEC and DJR across the two tasks.

## Discussion

The results of this study showed that the effects of tea consumption differed between the two tasks. Although the mental arithmetic task was based on the MATH algorithm [31], the original procedure was a machine-paced task in which problems were presented every 5 s. In this experiment, self-paced tasks were administered to calculate performance indices, such as the number of responses. In the self-paced task, participants could continue thinking until they reached an answer; thus, the correct rate was close to 100%, indicating a ceiling effect, in which a large proportion of participants achieved the maximum score, leaving little room for further improvement in performance. However, there were differences in response speed between the conditions, with a significant decrease in reaction time and a significant increase in the number of responses in the TEA condition compared to the WATER condition. Interestingly, in the TEA condition, there was a significant enhancement in these indices while maintaining a high percentage of correct responses. The SDS is a computer version of the Trail Making Test [33] and is a self-paced task. The correct rate was extremely high (approximately 96%) under all conditions, and there were no significant differences in any task performance indices between the conditions.

This observation necessitates the consideration of the potential influence of learning effects on task performance. Although counterbalancing the order of conditions is a standard method to control such effects, it was not feasible in this study because of the possible residual effects of tea components in the body. To minimize the influence of learning, participants practiced the task for 5 minutes more than twice before the experiment and performed the task again in the NONE condition. Thus, it is assumed that participants were sufficiently familiar with the task by the time of the WATER condition, suggesting that the learning effect may have already disappeared or been sufficiently weakened when comparing the WATER and TEA conditions. However, an experiment in which the conditions are randomized should be conducted in the future to confirm the extent to which the learning effect may have influenced these results.

For most SFF factors, there were significant increases after work compared with before under the NONE and WATER conditions. Importantly, in the TEA condition, all subjective fatigue factors—except for dullness—did not show significant increases after the task. Dullness, which did show an increase, is related to physical fatigue and consists of arm fatigue, lower back pain, hand or finger pain, leg fatigue, and shoulder stiffness. This is distinct from fatigue caused by mental activity. Therefore, the observed increase in dullness likely reflects physical strain from the task and not the effect of tea components. In contrast, whereas the feeling of drowsiness increased after the task in the NONE and WATER conditions, no significant change was observed in the TEA condition, and lower values were recorded. These results underscore an important distinction: although tea components did not appear to reduce physical fatigue, as reflected in the increased dullness score, they may have helped prevent the onset of mental fatigue and support the maintenance of arousal during cognitively demanding work. Regarding the NASA-TLX, the PD score was slightly higher in the WATER than NONE conditions during MATH. This result was similar to the Dullness Factor score in the SFF. However, although the Dullness score increased after the task in all sessions, the PD score in the TEA condition did not show significant changes compared to the NONE and WATER conditions. The NASA-TLX subscales, MD, PD, and TD, required a psychological evaluation of the task demands. The TEA condition was conducted in the third session on the same day, and based on the SFF results, the participants performed the task with a higher level of mental and physical fatigue. However, since the PD score did not increase in the TEA condition, it is speculated that the tea components might have influenced the psychological evaluation. The FR score increased in the WATER condition and decreased to the NONE level in the TEA condition. The FR scale assesses irritability and mental stress during the task, and a decrease in FR in the TEA condition may indicate the relaxation effect of green tea.

Regarding the flow score, the experience score was significantly higher in the TEA condition than in the NONE and WATER conditions, and the DJR score was significantly lower after the SDS. The Experience factor was the average score for complete concentration, forgetting oneself, losing track of time, and enjoying the task, which can be considered an indicator of the absorption level (Table 3). The high experience score in the TEA condition may be attributed to the arousal effects of tea components.

The flow state was expected to improve task performance; however, performance on the SDS task was not better than that in the TEA condition, possibly due to the ceiling effect on the correct response rate. It may also be related to the offsetting of performance scores caused by the mix of individuals whose performance increases (forming a lambda shape “Λ”) and those whose performance decreases (forming “V“ shape) in the WATER condition. These factors may have limited the detection of subtle differences. Future studies should consider using tasks or conditions that allow greater variability in responses.

Flow scores were higher for the SDS than for the MATH tasks. Both tasks are self-paced; however, the SDS may be more likely to induce a flow state as it has a shooting game-like element where participants find a target and hit it quickly under mild time pressure from the progress bar display. Table 4 summarizes self-reports of strengths and preferences for each task. Three participants reported not liking the MATH task despite being good at it, whereas the remaining participants indicated that they liked the SDS task but were not good at it.

**Table 4:** Number of participants in self-report tasks after the experiment.

		MATH	SDS
**Strengths**	Like	5	9
	Dislike	**3**	**0**
**Weaknesses**	Like	**0**	**4**
	Dislike	13	9
**Neither**		1	1

*Notes:* MATH: mental arithmetic task; SDS: sequence digit search task.

Although arithmetic tasks allow for the measurement of cognitive performance, their repetitive and highly structured nature may not necessarily facilitate the induction of a flow state as effectively as more creative tasks. The adapted version of the SDS used in this study has not been previously validated, which limits its comparability with other studies in the area. Moreover, the experience of flow may vary depending on individual preferences and cognitive strengths. Therefore, the rationale for task selection warrants further consideration, with factors such as creativity, novelty, individual variability, and the use of validated tasks being important focuses of future investigations.

One’s skills and challenges must be balanced at a high level to reach a flow state. We believe that the task’s requirements to induce a flow experience must be tackled continuously, even if one does not like it and has an element of enjoyment. Nevertheless, the TEA condition was the third trial in which the participants felt physical dullness, and a significantly higher flow score (experience) in this condition suggested that the SDS task included an element of enjoyment. Additionally, tea consumption helped maintain participant arousal.

The Trail Making Test, created by Reitan in 1944 [42], is part of the Army Individual Test Battery, and the test scores vary with age and education [43]. Participants were required to connect randomly displayed numbers or letters on a test form in ascending order using a pencil. This test comprehensively assesses attention, working memory, spatial exploration, processing speed, persistence, and impulsivity. In recent years, it has been increasingly regarded as a neuropsychological evaluation method for the aptitude to drive a car and can be used to evaluate higher brain dysfunction due to traumatic brain injury, mild cognitive impairment, relatively mild dementia, and relatively pure executive dysfunction, as typified by prefrontal cortex damage [33]. Additionally, by combining both numbers and letters, it is used as a neurophysiological indicator of the degree of brain damage and intelligence [44]. Performance on this test can be influenced by short-term memory ability, as participants must remember the locations of other numbers while searching for a target. However, a task in which all the numbers are shuffled after each correct answer, as in our experiment using a computer display, may be referred to as a vigilance task rather than a short-term memory task. According to the Dictionary of Psychology [45], vigilance is “the psychological state of being alert or concentrating attention, and the ability to maintain that state for a fairly long time.” Fukushima and Ibuka translated vigilance with a note that it is a state of arousal, alertness and attention. The term “vigilance” was used to describe a variable state of the central nervous system and the term “arousal” is almost synonymous with vigilance because it includes a state of the central nervous system [46].

Vigilance tasks are commonly associated with monitoring tasks, such as observing instruments in power and chemical plants or radar surveillance. These tasks are analogous to checking various meters in aircrafts and automobiles. Reduced vigilance as an impairing effect of the mental workload is considered a “fatigue-like status,” and is a state with reduced activation and detection performance, mainly associated with monitoring tasks offering only little variation. Reduced vigilance can be found in monitoring or inspection tasks, such as when monitoring radar screens or instrument panels [47].

The maintenance of arousal level (vigilance) and improvement in flow scores may be related to the elements of green tea. The present experimental findings are closely aligned with the theoretical model proposed by Reich et al. [25], who conceptualized caffeine as a chemical facilitator of flow states. Specifically, participants who ingested moderate doses of caffeine exhibited enhanced task engagement, significantly faster reaction times, and subjective experiences indicative of deep immersion, which are the hallmarks of the flow experience. These outcomes parallel the hypothesized neurochemical mechanisms, including increased dopaminergic “wanting” and noradrenergic arousal, both of which are considered central drivers of flow.

In addition, the maintenance of arousal level (vigilance) and inhibition of fatigue from tea consumption may be attributed to the combination of L-theanine and caffeine [10,12,48]. Notably, the observed behavioral and psychological effects emerged within a relatively short time frame following green tea ingestion. Given that previous studies have shown that L-theanine reaches peak plasma concentration approximately 48 minutes after oral administration of 250 mL of green tea [49], the immediacy of the effects observed in this study may not be fully explained by systemic absorption alone. One possible explanation is the contribution of aromatic components, which may exert psychophysiological influences via olfactory pathways. These possibilities raise important questions about the multimodal mechanisms of green tea’s action, including both ingestion and olfactory exposure, which warrant further investigation.

Although Reich et al. [25] emphasized physiological indices such as increased high-frequency component of heart rate variability (HF) and decreased stress perception during cognitively demanding tasks, the present study was limited to behavioral and subjective indicators, such as flow state and fatigue. Ongoing research is currently examining autonomic and neurophysiological responses, including the potential role of olfactory stimulation, and these findings will be reported in subsequent publications.

This study demonstrated that even a small amount of green tea consumed over a short period can help sustain mental functioning during cognitive tasks. These findings suggest the potential benefits of regular, moderate green tea intake in everyday work settings.

## Limitations

Although this study was limited to a sample of young male participants and caffeine tolerance varied among individuals, the findings may not be immediately generalizable to females or individuals in other races, or those in different age groups. It should also be noted that the number of participants was relatively small (n = 22), which may limit the statistical power to detect small effects. To address this concern, a post-hoc power analysis (α = 0.05, power = 0.80) was conducted using G*Power 3, which indicated that this sample size was sufficient to detect medium-sized effects (Cohen’s d ≈ 0.65). Given the exploratory nature of this study and the logistical constraints of a repeated-measures design, we aimed to balance statistical sensitivity with feasibility. Nevertheless, we acknowledge this limitation and recommend that future studies with larger and more diverse samples be conducted to confirm and extend these findings.

Moreover, due to the residual components of green tea, it was not possible to counterbalance the order of the three beverage conditions. Although conducting the study in a laboratory allowed for careful control of the experimental conditions, a limitation is that the results and conclusions may not necessarily be extrapolated to occupational settings. It should be noted that the peak physiological effects of tea consumption may not have been fully realized in some participants, as the assessments were conducted shortly after ingestion. Nevertheless, we believe that the consumption of green tea may prevent an increase in mental fatigue, maintain arousal levels, and even induce a flow state. In future research, it will be important to consider the effects of habituation to mental tasks and include a more diverse range of participants.

## Conclusion

This study demonstrated that the consumption of a small amount of green tea over a short period can positively influence cognitive task performance by maintaining arousal levels, preventing the progression of mental fatigue, and enhancing flow experience. These effects were observed even under minimal intake conditions, suggesting that green tea may serve as a practical and accessible means of supporting mental functioning and subjective well-being during cognitively demanding tasks.

The enhancement of the flow state is particularly noteworthy, as it indicates that green tea may not only suppress the increase in mental fatigue but also facilitate a more engaged and immersive task experience. These findings provide valuable insights into the subtle yet meaningful impact of naturally derived substances on psychological and physiological states.

Future studies should investigate the biological mechanisms underlying these effects, such as the roles of caffeine, L-theanine, or other components, and examine the generalizability of the results across different populations, types of mental tasks, and environmental conditions. Ultimately, these findings contribute to the growing body of evidence supporting the role of dietary components in promoting cognitive and emotional health.

## Supporting information

S1 Table Results of the component analysis of the beverages. (PDF)

S2 Table Summary of task performance (mean, SD, n, and range) for MATH. (XLSX)

S3 Table Summary of task performance (mean, SD, n, and range) for SDS. (XLSX)

S4 Table Summary of SFF scores (mean, SD, n, and range) for each condition. (XLSX)

S5 Table Summary of NASA-TLX scores (mean, SD, n, and range) for each task. (XLSX)

S6 Table Summary of the FEC and DJR scores (mean, SD, n, and range) for each task. (XLSX)
