# Supplementary material for: Green tea consumption rapidly enhances cognitive performance and flow state during mental tasks in healthy young adults
Source: PLoS One. 2025 Jul 10;20(7):e0328394. doi: 10.1371/journal.pone.0328394 (PMC12244585; doi:10.1371/journal.pone.0328394)
Supplement: S1 Table — (PDF) [file pone.0328394.s001.pdf]

S1 Table. Results of the component analysis of the beverages.

Theanine, caffeine anhydrous, and catechins are measured per 100g, and pyrazines are measured per 1L. A “<” in the table indicates that the content is less than the quantitative limit.

	Water	Green tea	Determination limit
Theanine	Not deleted	3mg	1.0mg
Caffeine anhydrous	Not deleted	0.013g	0.001g
Catechin	Not deleted	2.4mg	0.5mg
Epicatechin	Not deleted	1.4mg	0.5mg
Gallocatechin	Not deleted	12mg	0.5mg
Epigallocatechin	Not deleted	5.6mg	0.5mg
Epigallocatechin gallate	Not deleted	5.9mg	0.5mg
Gallocatechin gallate	Not deleted	6.5mg	0.5mg
Epicatechin gallate	Not deleted	1.1mg	0.5mg
Catechin gallate	Not deleted	1.1mg	0.5mg
Specific gravity (20°C)	－	1.001	
2-Ethyl-3,5-dimethylpyrazine [*)]	＜1.0μg	15μg	
Tetramethylpyrazine	＜0.5μg	＜0.5μg	
2,3-Diethyl-5-methylpyrazine	＜1.0μg	＜1.0μg	

Notes: In green tea and roasted green tea, the 2,3-diethyl-5-methylpyrazine peak overlapped with other peaks (isomers and presumed isomers). The overlapping peaks were subtracted from the quantification. In the case of water, a small amount of a peak that appeared to be derived from other ingredients was detected at the peak detection time of 2,3-diethyl-5-methylpyrazine; therefore, m/z 150 was used instead of m/z 121 for quantification.
